# Methotrexate alone and methotrexate combined with etanercept in treatment of rheumatoid arthritis

**DOI:** 10.1186/ar3661

**Published:** 2012-02-09

**Authors:** Sylejman Rexhepi, Mjellma Rexhepi, Blerta Rexhepi, Vjollca Sahatçiu-Meka, Vigan Mahmutaj

**Affiliations:** 1 Rheumatology Department, University of Prishtina, Prishtina, Kosova, 10000; 2 Private Clinic "Rheuma", Prishtina, Kosova, 10000; 3 Physical Medicine Department, University of Prishtina, Kosova, 10000

## Objectives

The aim of this study is to evaluate the efficacy and safety of methotrexate (MTX) alone and combined therapy of Etanercept (ETN) and methotrexate (MTX), in patients with rheumatoid arthritis (RA).

## Methods

Patients with RA were treated in combination with ETN (with doses of 25 mg subcutaneously twice weekly), with oral MTX (doses up to 20 mg weekly), and alone MTX (doses up to 20 mg weekly) in period of two years, in Rheumatology Department of Internal Clinic in Prishtina. Clinical response was assessed using American College of Rheumatology (ACR) criteria and the Disease Activity Score (DAS28) in 60 patients with RA. Radiographic changes were measured in the beginning and at the end of the study with Sharp Score.

## Results

Of total number of 60 patients (10 of them were males and 50 were females) with mean age of 57.63, 10 or 16.6% of patients were treated with combined therapy (ETN plus MTX) and 50 or 83.3% of patients with monotherapy (MTX). The group of combined therapy (ETN+MTX) after the treatment resulted with improvement of acute phase reactants as erythrocyte sedimentation rate (ESR) for the first hour (41.1 vs. 10.3 mm/hour) and C-reactive protein (CRP) (40.8 vs. 6 mg/liter) comparing to the group treated with MTX alone there were no significant changes (ESR: 45.7 vs. 34.3 mm/hour; CRP: 48 vs. 24 mg/liter). Before treatment the severity of the disease was high, where in group with combined therapy (ETN plus MTX) DAS28 was 5.32, and in the group with monotherapy of MTX DAS28 was 5.90. After 2 years of treatment we had significant changes in the results of DAS28, where in group treated with ETN plus MTX DAS28 was 2.12 ± 0.15, while in the group of patients treated with MTX DAS28 were 3.75 ± 0.39 (t = 13.03; df = 58; p < 0.0001). The group with combined therapy showed less radiographic progression comparing to the group of monotherapy (p < 0.05).

## Conclusions

According to our results we can conclude that ETN in combination with MTX reduced disease activity, slowed radiographic progression and improved clinical manifestations more effectively than MTX alone within period of 2 years. During the treatment, no serious adverse events were noticed with combination treatment of ETN and MTX.
